# Inequalities in Caries Experience Among Mongolian Children

**DOI:** 10.3390/ijerph16203892

**Published:** 2019-10-14

**Authors:** Tselmuun Chinzorig, Jun Aida, Upul Cooray, Tsengelsaikhan Nyamdorj, Soyolmaa Mashbaljir, Ken Osaka, Ariuntuul Garidkhuu

**Affiliations:** 1 Department of International and Community Oral Health, School of Dentistry, Tohoku University, Sendai 980-0872, Japan; tselmuunchnzo@gmail.com (T.C.); j-aida@umin.ac.jp (J.A.); upul.cooray.15@ucl.ac.uk (U.C.); osaka@m.tohoku.ac.jp (K.O.); 2 Graduate School, Mongolian National University of Medical Sciences, Ulaanbaatar 14210, Mongolia; tsengelsaikhan@mnums.edu.mn (T.N.); soyolmaa@mnums.edu.mn (S.M.); 3 School of Medicine, Department of Public Health, International University of Health and Welfare, 4-3 , Kozunomori, Narita-shi, Chiba ken 286-8686, Japan

**Keywords:** oral health inequality, caries prevalence, socioeconomic status, Mongolia

## Abstract

Although inequalities in dental caries have been well-reported, there is only one Mongolian study on the association between socioeconomic status (SES) and caries experience, which was published ten years ago. This study aimed to determine the dental health status of Mongolian children living in urban and suburban areas of Ulaanbaatar city and examine its association with income and parental educational attainment. An oral examination was conducted by dentists and caries were measured as deft/DMFT indices. A questionnaire including demographic characteristics and socioeconomic status was completed by their parents or caregiver. Parental educational attainment and household income were used as the measures of SES. The relative index of inequality (RII) and slope index of inequality (SII) were employed to examine the association between SES on deft and DMFT after adjusting for covariates. Dental caries prevalence (those with deft/DMFT > 0) was 89.3% among the total number of participants. The mean deft/DMFT values for age groups 1–6, 7–12, and 13–18 were 5.83 (SD = 4.37, deft), 5.77 (SD = 3.31, deft/DMFT), and 3.59 (SD = 2.69, DMFT), respectively. Rather than residence area and parental educational attainment, significant caries experience inequality was observed in relation to income (RII 0.65 95%, CI 0.52 to 0.82, SII −2.30, 95% CI −4.16 to −0.45). A prevention strategy for lower socioeconomic groups and building integrated oral health surveillance to monitor epidemiological trends for further evaluation of its progress is necessary.

## 1. Introduction

Oral diseases are highly prevalent globally, posing major public health issues [1,2,3,4] and considerably affecting the quality of life [5]. In spite of governments’ and nongovernmental agencies’ extensive investment in research and dental services, the trajectories of dental diseases are socially patterned, and are substantially more prevalent among poorer and disadvantaged populations [6,7,8,9]. The Global Burden of Disease 2015 Study reported a high prevalence of oral disease, with a 64% increase in disability-adjusted life years due to oral conditions throughout the world, and it has been reported that oral health has not improved for 25 years [10]. Social inequalities in oral health exist globally, irrespective of the state of development of countries, and a social gradient in oral health is also a universal phenomenon found at all points in the life course and in different population groups across the world [11]. Many previous studies have reported the presence of a social gradient by showing that low socioeconomic status (SES) groups exhibit worse oral health and a higher prevalence of caries compared to higher SES groups [12,13]. However, studies on oral health inequalities in low and middle income countries are relatively scarce.

Oral health inequalities are known as “A Canary in the Coalmine” [14] due to their profound prevalence from an early stage of life, while leading the front line in inequalities in other diseases. Regarding caries experience in childhood, it is well-known that one of the determinants is SES [15]. Indicators of SES, such as household income and educational level, are important factors that have an impact on caries experience [16,17].

Dental caries is highly prevalent in Mongolia. A survey conducted by the School of Dentistry, Mongolian National University of Medical Sciences, demonstrated that 93.2% of the urban population suffers from dental caries [18]. In fact, dental caries in children is the most prevalent condition among all age groups in urban areas [19,20,21]. It was reported that in the age group of 3 to 6 years, the carries prevalence was 75.5% and 78.5% and the deft score was 4.9 and 5.3 in 1993 and 1996, respectively. In 2005 [22], the caries prevalence was estimated as 96.1% in the same age group. The Government of Mongolia approved their second “National Oral Health” program in 2006, which included two phases: 2006–2010 and 2011–2015. This was development with the expectation of a reduction of caries prevalence of up to 78.0% to 80.1% among 5 to 6 year olds, 60.0% to 62.0% among 12 year olds, and 70.0% to 71.6% in the population, based on the World Health Organization’s (WHO) recommendation [23].

Despite the national program, dental caries is highly prevalent in Mongolia [18,24] and the prevalence of dental caries has remained steady for the past 20 years [24]. Studies on caries inequalities among Mongolian children are limited. Although a few studies have been conducted on caries prevalence, recently, no study has examined social inequalities in dental caries in urban areas. To the best of our knowledge, the only study published internationally was conducted ten years ago, in 2009 [21]. Therefore, this study aimed to determine social inequalities in dental caries among children aged 1 to 6, 7 to 12, and 13 to 18 years old living in suburban and central districts of Ulaanbaatar city.

## 2. Materials and Methods

The data used in this study derived from a cross-sectional study consisting of a total of 320 participants aged 18 years or less in Ulaanbaatar, the capital city of Mongolia, which consists of nine administrative districts. Considering the regional SES, districts were categorized into two types: urban and suburban. Districts within the areas were chosen by a simple random sampling method: in total, 121 participants from Bayangol (child population = 76,681) and Sukhbaatar (child population = 47,203) districts as central areas, and 199 participants from Nalaikh district as a suburban area (child population = 12,276 [25]), were included. Children in public kindergartens and schools were asked to participate in a questionnaire survey and dental examination. 

Inclusion criteria were an age ranging from 1 to 18, both sexes, and no ethnic distinction. The exclusion criteria were parents/caretakers who did not allow the participation of children in the study. Written informed consent was obtained from all the parents or guardians. There were no instances of declined consent; however, some children could not be examined due to the absence of consent. This study was approved by the Institutional Review Board (IRB) of the Mongolian National University of Medical Sciences and conducted in accordance to Ulaanbaatar City governor’s request (#02-03/626) as preliminary research within the “Healthy Tooth-Healthy Child” National Program Framework. The study followed the “Strengthening the Reporting of Observational Studies in Epidemiology” (STROBE) guideline. 

## 3. Survey and Dental Examination

A questionnaire survey and dental examination were conducted in this study. The questionnaire consisted of information on a child’s background, such as sex, age, residence area, daily brushing frequency, dental visits, and caretaker’s age, as well as socio-economic information, such as family income and parental educational attainment. Based on the National Statistics Office of Mongolia, the income was categorized into four groups [26]. Income was used as an indicator for SES and the Mongolian tugrik was converted to the US Dollar. For the dental clinical examination, two trained dentists from the School of Dentistry, Mongolian National University of Medical Sciences (MNUMS), performed the procedure in kindergarten and public school classrooms using a disposable dental mirror with light-emitting diode (LED) light and a dental probe. The diagnostic criteria for caries followed the recommendation of the WHO, and filled but caries-experienced teeth were recorded as decayed [27]. Caries experience was measured with the deft/DMFT index. For deciduous teeth among younger children, caries experience was recorded as deft, where “e” stands for exfoliated/extracted teeth. For permanent teeth and mixed dentition among older children, deft/DMFT was applied. A tooth was recorded as decayed (d/D) when a lesion had a definite cavity, undermined enamel, or a detectably softened floor or wall. A tooth was recorded as filled (f/F) when it was permanently filled without caries. A tooth was recorded as missing (M) when it was extracted due to caries. A standard two examiners from the School of Dentistry, Mongolian National University of Medical Sciences, pre-trained and calibrated by a “gold standard” examiner, performed dental examinations of children (Kappa = 0.92). As the two examiners had extensive clinical experience, intra-examiner agreement was not assessed. After the dental examination, the child’s oral health report was given to the parents of each child. No dental treatment was provided on site. Parents could seek further dental treatment by their own cost if necessary. 

## 4. Statistical Analysis

To measure socioeconomic inequalities and gradients in caries experience, absolute and relative measures of inequalities, the slope index of inequality (SII), and the relative index of inequality (RII) were used [28,29]. Both RII and SII were calculated using the method suggested by Mackenbach and Kunst [30]. The SII estimated the absolute predicted difference in caries experience between the lowest and highest SES. Conversely, RII estimated the risk ratio for caries between the highest and lowest SES. Unfortunately, caries experience in this study was only available as combined deft/ DMFT. Therefore, as a sensitivity analysis, we applied age-stratified analysis by two age groups, those aged 1 to 9 years and 10 to 18 years, to reflect the difference of deft and DMFT: Caries among children aged 1 to 9 years was considered to mainly occur in primary dentition and caries among children aged 10 to 18 years old was considered to mainly occur in permanent dentition. SES variables, household income, and parental educational attainment were ordered from low to high. SII of 0 or RII of 1 shows that there is no consistent relationship between caries experience and SES. A negative SII or RII with a value of less than 1 indicates absolute and relative inequalities; caries experience is lower in the children with higher SES. The ridit score for estimating SII and RII was calculated by the RIIGEN command in STATA [31]. Using the ridit score and continuous caries experience measurements, the ratio of the mean by Poisson regression was considered as RII and the beta coefficient by linear regression was considered as SII. Multiple imputation by the chained equations method was applied to treat missing data, and 20 imputed datasets were generated. The ridit score, RII, and SII were calculated for each of the 20 datasets and RII and SII were integrated. Significance was determined at α = 0.05 in all of the tests. The results of the oral examination and questionnaire surveys were analyzed using STATA MP 15.0 (Stata Corp., College Station, TX, USA). 

## 5. Results

This study included a total of 320 participants and there were 110 boys (34.4%). Dental caries prevalence was 89.3% among the participants. The distribution of participants’ characteristics by caries experience is shown in Table 1. No significant difference in caries experience was shown by the current area of residence. For the dental visit category, 23.4% (N = 75) of the participants responded as visiting dental clinics in order to prevent possible dental decay. However, the caries deft/DMFT did not differ between those who visited dental clinics for preventive measurement and those who visited them after experiencing toothache. The mean deft/DMFT for the participants of 1–6, 7–12, and 13–18 were 5.83 (SD = 4.37), 5.77 (SD = 3.31), and 3.59 (SD = 2.69), respectively. The mean deft/DMFT was higher when the caretaker’s age was older: 20–29, 30–39, 40–49, and above 50 years old groups produced results of 5.73 (SD = 3.75), 5.52 (SD = 4.01), 5.52 (SD = 3.77), and 7.01 (SD = 4.70), respectively. Participants with a higher monthly household income had lower caries deft/DMFT compared to those from a lower household income, with values of 4.35 (SD = 3.04) versus 6.69 (SD = 4.36), respectively. 

The inequalities in caries experience according to household income and parental educational attainment are presented in Table 2. The RII, relative measurement of inequality, showed the socioeconomic inequality for household income for both younger and older children, as well as non-stratified analysis. After adjusting for all covariates, RII for the whole sample was 0.65, 95% CI 0.52 to 0.82. For the age-stratified analysis, RII values for younger and older children were 0.62, 95% CI 0.48 to 0.81 and 0.53, 95% CI 0.32 to 0.88, respectively. Similar to the results of RII, there was significant absolute income-related inequality. SII for both younger and older children was −2.30, 95% CI −4.16 to −0.45, after adjusting for all covariates. Although statistical significance was not observed among older children, for which the number of analyzed individuals was very small, the 95% CIs were marginal and the direction of inequality was consistent with other age groups. There was significant absolute income inequality among younger children, with results of SII −2.80, 95% CI −5.11 to −0.48.

## 6. Discussion 

This study aimed to determine the current burden of dental caries and to identify social inequalities in the caries prevalence among Mongolian children living in urban and suburban areas. The results of this study showed that the prevalence of caries in Mongolian children is still high and has not significantly changed since 1993. There was no significant difference in caries experience among participants in urban and suburban districts. More than half of the participants (62.8%) had visited a dental clinic after having a toothache. For those who had visited a dental clinic in order to prevent problems and those who had approached a clinic after having certain dental pain, the caries deft/DMFT was almost the same, with values of 5.67 (SD = 4.12) and 5.33 (SD = 3.64), respectively. The mean deft/DMFT was higher when the caretaker’s age was increased. Children in families with a lower income had significantly higher caries experience. However, parental education did not show a significant association with deft/DMFT in children.

Studies have indicated that living in a rural area is one of the disadvantages in accessing dental health services due to a lack of availability of necessary facilities and dentists [32,33,34]. On the contrary, previous studies in Mongolia have demonstrated that caries levels among children and adolescents are significantly higher in urban areas than in rural areas [19]. To our knowledge, no study exists on caries disparity among those who reside in urban and suburban areas. However, the present analysis did not show any significant difference in caries status between the urban and suburban districts of Ulaanbaatar city. According to a previous study on the School-Based Fluoride Mouth Rinse (S-FMR) program, this program was associated with lower DMFT [35]. Therefore, to reduce the persistent high caries experience, utilizing S-FMR programs in Mongolia as a population strategy for dental caries prevention might be effective. 

A large systematic review on the social gradient of caries experience showed that education and income were significantly associated with caries [3,9,36,37]. Conversely, our study showed that parental educational attainment has no association with dental caries experience in children. This result was consistent with a previous Mongolian report which showed that lower educational attainment is not a risk factor for dental caries experience [20] However, global evidence suggests that higher educational attainment is associated with better health outcomes. In order to reflect the educational attainment of individuals and their offspring’s health, the Mongolian education system might have to integrate health education into its agenda.

Among the social and demographic factors, income has a stronger association with caries experience than living area and parental education attainment. From the perspective of Universal Health Coverage (UHC), income-related barriers for accessing dental health services should be determined and eliminated. For instance, the National Health Service (NHS) in the UK provides free dental care for those who are under 18 years old [38]. In Japan, the National Health Insurance (NHI) covers 70% of the total cost of care, including dental clinics [39]. In Mongolia, health insurance covers dental basic treatment, but it only applies for children referring to state dental clinics. However, approximately 92% of all dental services are provided by private clinics in Mongolia. Although, in 2019, the National Program for Healthy Tooth-Healthy Child was launched, with the aim of being implemented for three years [40,41], dental insurance design to offer certain coverage at a preventive level and basic dental treatment to be used for dental private practices is needed. In order to achieve UHC, developing a UHC package that meets the needs of those who consider cost as a barrier to accessing dental health services is required [42]. This includes advocacy in oral health among policy makers, the media, civil society organizations, and oral health professionals. 

## 7. Strengths and Limitations

The strength of this study is that it is a relatively rare study in Mongolia in terms of examining social inequalities in caries experience. The only study examining the association between caries experience and SES was published in 2009, which is out of date. Compared to all previous studies, most of which are on caries prevalence, this study examines other associations with the burden of dental caries in urban and suburban areas. We could not access inter- and intra-examiner agreement for caries measurements. If there was a larger random variation of caries diagnosis between dentists, 95% CI of the observed association between SES might be widening.

There are also several limitations. The study questionnaire was not filled out by a parent exclusively, but by a caretaker, such as a grandparent, which may have led to information bias. Collected data had several missing values. Due to the small sample size, it may not have an adequate statistical power to elicit significant associations. In addition, due to the limited sampling areas, generalizability of the present results to the whole of Mongolia was limited. In addition, we could not distinguish between deft and DMFT. Therefore, we applied age-stratified analysis because caries experiences in primary teeth and permanent teeth were considered to depend on age. Regarding the recoding of missing teeth for primary dentition (e teeth), our data could not determine whether primary teeth were extracted due to caries or natural exfoliation. If natural exfoliation was included in the deft index, this recode does not exactly reflect pure caries experience. This bias could cause an underestimation of the association between SES and deft. Even in this is an underestimation, our analysis showed a statistically significant social gradient in childhood caries experience. Therefore, the association was considered to be robust. 

## 8. Conclusions

Residence area and parental educational attainment were not associated with caries experience among Mongolian children. Income inequalities in childhood dental caries experience were observed. Strengthening the childhood caries prevention strategy for lower socioeconomic groups and building integrated national oral health surveillance to monitor epidemiological trends for further evaluation of its progress are necessary. 

## Figures and Tables

**Table 1:** Caries experience distribution by participants’ characteristics.

	N	%	Caries Prevalence	Mean Deft/DMFT ± SD
**Sex**				
Male	110	34.4	90.9	5.86 ± 3.73
Female	116	36.4	86.2	5.19 ± 4.01
Missing data	94	29.4	91.5	5.41 ± 3.99
**Age group**				
1–6 years old	169	52.8	87.0	5.83 ± 4.37
7–12 years old	105	32.8	94.3	5.77 ± 3.31
13–18 years old	46	14.4	87.0	3.59 ± 2.69
**Residence area**				
Urban	121	37.8	90.9	5.32 ± 3.73
Suburban	199	62.2	88.4	5.59 ± 4.02
**Parental education attainment**				
Secondary education	138	43.1	91.3	5.63 ± 4.04
Tertiary education	163	50.9	88.3	5.43 ± 3.75
Missing data	19	6.0	84.2	4.95 ± 4.39
**Monthly household income (Mongolian tugrik converted to US dollar)**				
<$189	61	19.1	95.1	6.69 ± 4.36
$190 to $379	114	35.6	89.5	5.43 ± 3.83
$380 to $569	70	21.9	85.7	5.34 ± 4.00
≥$570	38	11.8	80.6	5.13 ± 3.66
Missing data	37	11.6	94.6	4.35 ± 3.04
**Caretaker’s age group**				
20–29 years old	63	19.7	79.4	5.73 ± 3.75
30–39 years old	145	45.3	90.3	5.52 ± 4.01
40–49 years old	61	19.1	91.8	5.52 ± 3.77
Above 50 years old	22	6.9	95.5	7.01 ± 4.70
Missing data	29	9.0	82.7	3.48 ± 2.61
**Daily brushing frequency**				
At least once a day	83	25.9	83.1	4.94 ± 3.77
Twice a day	150	46.9	91.3	5.72 ± 3.95
Three times a day	34	10.6	94.1	6.91 ± 3.78
Missing data	53	16.6	90.6	4.79 ± 3.87
**Dental visits**				
Visits after toothache	201	62.8	87.1	5.67 ± 4.12
Visits to prevent	75	23.4	90.7	5.33 ± 3.64
Missing data	44	13.8	97.7	4.93 ± 3.33

**Table 2:** Relative (relative index of inequality (RII)) and absolute (slope index of inequality (SII)) inequalities in dental caries experience according to monthly household income and parental educational attainment ^a^.

	RII [95% CI]				SII [95% CI]			
	Unadjusted Model		Adjusted Model ^b^		Unadjusted Model		Adjusted Model ^b^	
**Children aged 1 to 9 years (*N* = 236)**								
Monthly household income	0.71 *	[0.58; 0.86]	0.62 *	[0.48; 0.81]	−2.04 *	[−3.94; −0.15]	−2.80 *	[−5.11; −0.48]
Parental educational attainment	0.78 *	[0.62; 0.98]	0.93	[0.70; 1.25]	−1.50	[−3.70; 0.71]	−0.45	[−3.11; 2.20]
**Children aged 10 to 18 years (*N* = 84)**								
Monthly household income	0.57 *	[0.36; 0.90]	0.53 *	[0.32; 0.88]	−2.09	[−4.29; 0.12]	−2.27	[−4.64; 0.10]
Parental educational attainment	0.91	[0.54; 1.54]	0.97	[0.55; 1.69]	−0.33	[−2.92; 2.25]	−0.09	[−2.84; 2.66]
**All children aged 1 to 18 years (*N* = 320)**								
Monthly household income	0.71 *	[0.60; 0.86]	0.65 *	[0.52; 0.82]	−1.80 *	[−3.37; −0.23]	−2.30 *	[−4.16; −0.45]
Parental educational attainment	0.95	[0.77; 1.18]	1.10	[0.86; 1.43]	−0.25	[−2.04; 1.53]	0.56	[−1.52; 2.64]

* *p* < 0.05. ^a^ Multiple imputation was applied to treat missing data. ^b^ Adjusted for age, sex, residence area, caretaker’s age group, daily brushing frequency, and dental visits.
